# Chitosan, Chitosan Derivatives, Polysaccharides and Their Applications—2nd Edition

**DOI:** 10.3390/molecules31050761

**Published:** 2026-02-25

**Authors:** Agnieszka Ewa Wiącek

**Affiliations:** Department of Interfacial Phenomena, Faculty of Chemistry, Maria Curie-Skłodowska University, Maria Curie-Skłodowska Sq. 3, 20-031 Lublin, Poland; agnieszka.wiacek@mail.umcs.pl

**Keywords:** polysaccharide, chitosan, biopolymer composites, medical therapy, tissue engineering, food industry

## Abstract

The field of polysaccharide systems, primarily chitosan and its derivatives, is a constantly evolving area of science. It offers significant promise for many practical applications due to the numerous beneficial properties of these biopolymers. This trend is confirmed by the next special issue of the journal “Molecules”, titled “Chitosan, Chitosan Derivatives, Polysaccharides and Their Applications—Second Edition.” This issue is devoted to the latest advances in polysaccharide research conducted in leading laboratories worldwide, with a particular emphasis on new perspectives and the latest, interesting applications in the food, agricultural, medical, biotechnological, and tissue engineering industries. The manuscripts included in this collection focus on research data regarding the production of these systems, their modification, and the application of highly specialized methods to advance science and, above all, improve health and quality of life. This special issue is organized and thematically divided into several areas related to medical and food applications, the combination of polysaccharides with other substances such as polymers, and theoretical studies. Several of the chapters conclude with a review article or even several. All articles (24 in total) included in this special issue are of high quality and represent the latest trends in research on innovative polysaccharide systems.

## 1. Chitosan-Based Systems in Medical Applications

### 1.1. Medical Therapy

Biopolymer drug delivery systems are designed to increase the bioavailability and therapeutic efficacy of poorly soluble compounds of biological importance. In the article titled “A Biopolymeric Dextran-Chitosan Delivery System for Controlled Release of Antioxidant and Anti-Inflammatory Compounds: Lignin and Curcumin”, Cucu et al. discuss studies combining various components (dextran, carboxymethyldextran, chitosan) to develop materials with controlled release of lignin and/or curcumin and antioxidant/anti-inflammatory properties [Contribution 1]. A mechanical evaluation showed that these three-component chitosan/dextran-based systems exhibited the highest tensile strength in the diametrical direction, significantly greater than the two-component systems. The authors explained that this is a result of strong hydrogen bonding/interactions between curcumin and the matrix components. Curcumin release kinetics, using a model based on the Weibull equation, showed that the multicomponent systems tested exhibited a slower release rate, thus providing prolonged bioactivity. An additional advantage of the tested systems is the retention of significant antioxidant and anti-inflammatory activity, at 60% and 70%, respectively, making them strong candidates for biomedical applications requiring prolonged therapeutic action, such as the treatment of osteoarthritis [Contribution 1] [1,2].

Triple-negative breast cancer is considered one of the most aggressive cancers, with a mortality rate of 40% within just five years of tumor appearance. Furthermore, it has limited treatment options and a poor prognosis. Therefore, researchers are working to develop new therapeutic strategies. One of these is photodynamic therapy (PDT) as an alternative to targeted therapy. Uddin and co-workers [Contribution 2] conducted a comparative analysis of the efficacy of PDT method and determined the induction of cytotoxicity at low laser power, as well as intracellular singlet oxygen production and cellular uptake for both treatment methods. Toxicity in normal breast cells was also assessed in the dark. Despite the low laser power and rose bengal (RB) nanoparticle concentration, the cells showed a significant decrease in viability. Additionally, RB nanoparticles demonstrated higher singlet oxygen production and uptake by cancer cells than RB solutions [Contribution 2] [3,4].

### 1.2. Tissue Engineering

Salim Hamidi et al. [Contribution 3] contributed a study titled “Design and Evaluation of a Cross linked Chitosan-Based Scaffold Containing Hyaluronic Acid for Articular Cartilage Reconstruction”, in which they presented a way to design freeze-dried hydrogels based on chitosan (CHT) and hyaluronic acid (HA) cross-linked with oxidized maltodextrin (MDo) to obtain spongy porous scaffolds [Contribution 3]. The physicochemical, mechanical, hygroscopicity/swelling and degradation properties of cross-linked chitosan-based scaffolds were analyzed to investigate the resistance of the scaffolds to enzymatic and acidic degradation. Studies of the resulting scaffolds demonstrated reduced swelling, slower degradation rates, and increased stiffness, confirming their suitability as a cross-linking agent. Furthermore, scaffolds containing ciprofloxacin demonstrated the ability to deliver therapeutic agents, as confirmed by microbiological studies. In conclusion, the authors concluded that such scaffolds, characterized by increased stability, functionality and mechanical properties, while being devoid of toxicity, may prove promising in cartilage tissue engineering [Contribution 3] [5,6].

Another article by Jurak and co-workers [Contribution 4] describes systems with natural polysaccharides for applications in tissue engineering, drug delivery and wound healing. The authors studied the interactions between polysaccharides (chitosan and/or hyaluronic acid), drugs (a nonsteroidal anti-inflammatory naproxen) and biological membranes. A 1,2-dipalmitoyl-*sn*-glycero-3-phosphatidylcholine (DPPC) monolayer was used as a model membrane in the Langmuir monolayer technique combined with Brewster angle microscopy (BAM) studies. The obtained compression/adsorption isotherms and morphology images allowed to determine the effect of the subphase type on the behavior of monolayers in the absence/presence of NAP, as well as their elasticity, morphology and stability as a function of time. The mode of interactions proposed by the authors can regulate the efficiency of drug delivery systems, meaning that it holds importance for living organisms in processes of pain relief and wound healing [Contribution 4] [7,8].

### 1.3. Biomedical Composites

Chitosan, a natural biopolymer from the polysaccharide group, exhibits antimicrobial and strong chelating properties, making it an ideal matrix for obtaining bioactive composites with the addition of metal ions. In the article titled “From *Hermetiaillucens* Pupal Exuviae to Antimicrobial Composites: Metal Nanoparticles Synthesized by Laser Ablation in Sustainable Chitosan Matrices” [Contribution 5], silver and copper nanoparticles were synthesized via the laser ablation (LAL) of metallic targets in commercial solutions of chitosan or natural chitosan produced from the exuviae of *Hermetiaillucens* pupal. Physicochemical characterization was performed using UV–vis spectroscopy, TEM microscopy, and FTIR spectroscopy (nanoparticle size, polymer/metal nanoparticle interactions). Antibacterial tests demonstrated the efficacy of Ag- or Cu-based composites against both *Escherichia coli*/*Micrococcus flavus* (with MIC, minimum inhibitory concentration, values equal to 0.006 g/L or 0.003 g/L, respectively). While Ag-based systems exhibited antibacterial activity in both colloidal and film forms, Cu-based systems exhibited this type of activity only in colloidal form. Additionally, swelling tests of films indicated excellent high water absorption capacity, over 200%, unaffected by nanoparticle integration. These results highlight the potential of metal–chitosan composites obtained using the LAL method, particularly those based on natural chitosan, as sustainable and effective antimicrobial materials for biomedical/environmental purposes [9,10].

The article by Rykowska et al. [Contribution 6] in this special issue is a review article describing solutions useful in medical therapies. One of these concerns therapies used in eye diseases. Drug delivery in this area remains a clinical challenge due to protective barriers in the structure and behavior of the eye (reflex blinking and regular tear fluid exchange). These factors significantly limit the bioavailability of topical medications, reducing the therapeutic efficacy of conventional formulations (eye drops, ointments/suspensions), particularly in the treatment of chronic ocular diseases (dry eye syndrome, age-related macular degeneration or diabetic retinopathy) [Contribution 6] [11,12].

The authors described research on the properties of drug-eluting contact lenses, which may be a promising solution for localized and controlled drug release directly onto the ocular surface while ensuring sustained release. This review differs from similar studies in that it focuses primarily on systems using biodegradable polymers. Particular emphasis is placed on describing the challenges and successes in designing lenses using natural and synthetic polymers characterized by high biocompatibility, highly flexible degradation kinetics, and controlled drug release [Contribution 6] [11,12].

The review by Rykowska and co-workers focuses on the selection criteria for polymer/biopolymer matrices, strategies for drug incorporation, and key factors influencing their release profiles. Moreover, the studies described in the review highlight innovative methodologies and/or therapeutic approaches to developing next-generation ocular drug delivery (bio)polymer-based systems and provide a useful, up-to-date, and comprehensive source of information for researchers [Contribution 6] [11,12].

The next review study in this research area, titled “From Molecules to Mind: The Critical Role of Chitosan, Collagen, Alginate, and Other Biopolymers in Neuroprotection and Neurodegeneration”, examines the use of various (natural and synthetic) biopolymers in neuroprotective systems that may play a potential role in the treatment of neurodegeneration. Among such biopolymers, be key therapeutic agents for neurodegenerative disorders are chitosan, fish collagen/gelatin, and alginate. It is well known that the treatment of these disorders poses significant challenges due to the complexities of delivering drugs to the central nervous system [Contribution 7] [13,14].

In this article, Kruczkowska et al. discuss the main mechanisms of neurodegeneration, which provides a starting point for understanding the interactions of biopolymers with neural tissue. The authors summarize the latest studies describing the effectiveness of biopolymer-based drug delivery systems in crossing the blood-brain barrier and exerting neuroprotective effects [Contribution 7]. Biopolymer-based approaches, including promising materials such as lignin, poly(lactic-co-glycolic acid), and bovine serum albumin, offer unique advantages in both neuroprotection and drug delivery. These well-studied systems open up new avenues for the treatment of neurodegenerative diseases. This review organizes the current knowledge on biopolymer-based therapeutic strategies in this field of medicine and identifies potential risks and challenges [Contribution 7] [13,14].

## 2. Chitosan-Based Systems in Food/Plant Applications

Replacing chemicals with eco-friendly biological products is becoming increasingly popular in food/plant application, mainly in product protection. Due to its biocompatibility, biodegradability, and bioactivity, chitosan is an effective agent against various food/plant diseases. The key aim of this chapter is to evaluate chitosan-based systems as potential biopesticides, anti-insect agents, and antimicrobial materials. Moreover, such systems can act as elicitors. Elicitors are chemical substances that stimulate plants to activate their natural defense mechanisms. They can act like a “vaccine,” triggering the production of phytoalexins and increasing resistance to fungi, bacteria, and viruses.

The purpose of the study described in the next article, “Chitosan as an Antimicrobial, Anti-Insect, and Growth-Promoting Agent for Potato (*Solanum tuberosum* L.) Plants”, by A. Steglińska et al., was to evaluate different kinds of chitosan as a potential biopesticide for potato plants. Chitosan of various molecular weights, high, medium and low, was tested for its antibacterial/antifungal properties against the strains of mold/bacteria responsible for potato diseases [Contribution 8]. The potential cytotoxicity of chitosan was assessed on various cell lines: Sf-9 insect cell line, HaCaT human keratinocytes, and Caco-2 human colon carcinoma. The bioprotective activity of chitosan was also assessed *in situ* on potato tubers. Chitosan inhibited the growth of selected phytopathogens, and the most active was chitosan of medium molecular weight. Polysaccharide formulation in lactic acid was characterized by low toxicity to human cells and high toxicity to Sf-9 cells. The selected chitosan formulations also have a positive effect on stem/root growth, gas exchange, and chlorophyll index in potato plants. On this basis, the authors proposed the use of chitosan-based systems as functional biopesticides to protect potatoes against phytopathogens [Contribution 8] [15,16].

This next review, titled “Chitosan as an Elicitor in Plant Tissue Cultures: Methodological Challenges”, by R. Chowdhury, summarizes methodological approaches to using chitosan-based systems for elicitation in plant tissue culture and highlights specific features of these procedures. It is well known that chitosan, a cheap biodegradable/biocompatible biopolymer derived from chitin, which exhibits diverse biological activity and is eco-friendly, represents a promising agent in plant tissue culture [Contribution 9]. Recent studies summarized in this review have highlighted its role as a natural elicitor that can promote plant growth, seed germination, and the biosynthesis of secondary metabolites *in vitro*. In plant tissue cultures, it acts as a mimic of pathogen attack and activates pathogenesis-related proteins, inducing the production of secondary metabolites. *In vitro* tissue culture is a helpful approach to studying elicitation mechanisms, but it is important to note that this methodology is neither simple nor uniform due to differences in chitosan’s physicochemical properties (e.g., origin, molecular weight, or degree of deacetylation), which directly affect solubility and the processes involved [Contribution 9] [17,18].

## 3. Chitosan–Polymer Compositions and Their Applications

The proposed chapter gives descriptions simple and environmentally friendly polysaccharide-polymer composites, which are useful in many fields.

Recently, eco-friendly flame retardants have become a popular alternative to traditional ones, which typically contain toxic halogens. B. Podkościelna et al. reported on the use of chitosan as an eco-friendly flame retardant [Contribution 10]. The aim of this study was to synthesize and analyze PVC blends using aluminum dibutyl phosphate or chitosan (at concentrations of 10–50 wt%), derived from the natural biopolymer chitin, as additives. These modern phosphorus-, nitrogen-, or silicon-based additives minimize harmful gas emissions during combustion, making products more environmentally friendly and safer for human health. The chemical structure and physicochemical properties of the newly synthesized hybrid composition were investigated using ATR/FTIR spectroscopy and SEM-EDX analysis. Additionally, dry composites based on PVC with the addition of stabilizer, plasticizer and chalk were prepared, which allowed researchers to obtain homogeneous materials for the assessment of fire resistance, thermal stability (DSC, TGA) and mechanical strength [Contribution 10] [19,20].

In the next study, Bukharbayeva et al. proposed a method for the synthesis of polymer–inorganic composites, which involved the modification of zinc oxide or montmorillonite with chitosan, followed by palladium immobilization. The structure and physicochemical properties of the obtained ternary composites were characterized using IRS, TEM, XPS, SEM, EDX, XRD, and BET methods [Contribution 11]. The interaction of chitosan with zinc oxide led to the deprotonation of polyelectrolyte amino groups and deposition on the surface of ZnO. The immobilization of Pd on modified surface occurred via the hydrolysis of [PdCl_4_]^2−^. Protonated amino groups of chitosan interacted with negative sites of montmorillonite, forming a positively charged two-component composite through electrostatic force. According to TEM studies, the presence of Pd nanoclusters with nanoparticles 3–4 nm in size were observed on different chitosan/ZnO sites. For hybrid chitosan/montmorillonite surface, Pd nanoparticles with sizes of 2 nm were evenly distributed on the support. It was shown that the efficiency of hybrid with montmorillonite is higher than analogous with ZnO, which can be explained by the formation of smaller Pd particles. Additionally, the mechanism of hydrogenation over an optimal 1%Pd–chitosan/montmorillonite catalyst was proposed [Contribution 11] [21,22].

In a subsequent article, Pellis and co-workers described the physicochemical properties and methods for improving the durability of the inhibitor chosen to develop new acrylic coatings for bronzes. Various outdoor statues are constantly exposed to atmospheric conditions and reactive compounds in the atmosphere that can interact with their surfaces. To avoid this, a common method is to apply corrosion inhibitor coatings. However, a significant limitation of these inhibitors is their gradual loss of properties over time [Contribution 12]. Methyl-β-cyclodextrin was chosen as the inhibitor carrier due to its ability to form complexes with organic substances. The formation of cyclodextrin-based complexes was confirmed using Fourier transform infrared spectroscopy, X-ray diffraction, and nuclear magnetic resonance spectroscopy. The prepared acrylic coatings at various concentrations were subjected to thermal aging, and the oxidation process of the corrosion inhibitor was monitored every 24 h. The obtained complexes proved to be promising candidates for the development of coatings with increased stability and longer retention times [Contribution 12] [23,24].

Further research on anticorrosion formulations was described in the article “Chitosan–Surfactant Composite Nanocoatings on Glass and Zinc Surfaces Prepared from Aqueous Solutions” by P. Márton et al. Hydrophobic coatings made of chitosan–surfactant composites with an average thickness of approximately 400 nm on glass–zinc substrates can act as anticorrosion formulations. Two basic surfactants (sodium dodecyl sulfate, SDS, or sodium dodecylbenzenesulfonate, SDBS) were used in this study. The coatings were prepared as follows: surfactants were mixed with aqueous chitosan solutions before coating application, or previously deposited chitosan coatings were impregnated with aqueous surfactant solutions. In the case of mixed coatings, the lower surface tension of the solutions (40–45 mN/m) corresponded to more hydrophobic (80–90°) coatings. The hydrophobicity of the impregnated coatings was particularly significant in the case of SDS and SDBS, with values of 88° and 100°, respectively [Contribution 13] [25,26].

AFM studies revealed a slight increase in roughness for the most hydrophobic coatings. Surfactant accumulation within the layer was significant in the impregnated samples, as confirmed by X-ray photoelectron spectroscopy. The authors demonstrated better barrier properties of the samples and a significantly lower swelling index in a water vapor atmosphere for the impregnated coatings than for the native coatings (approximately 75%). The authors concluded that good coating properties of the tested layers require both surface hydrophobicity and favorable bulk properties.

The paper by Filipkowska and Jóźwiak presents studies of the sorption of dyes (anionic Reactive Black 5, RB5, and Reactive Yellow 84, RY84) from single solutions or dye mixtures onto chitosan sorbents [Contribution 14]. The authors determined the effect of pH on sorption efficiency, anionic sorption equilibrium time, and sorption capacity for individual dyes and their mixtures, as described by the Langmuir equation. It was found that the sorption process for both dyes was most effective at acidic pHs in the range of 3–4, depending on the type of chitosan system. The obtained constants allowed us to determine the affinity of the tested dyes for three different sorbents and their sorption capacity [Contribution 14] [27,28].

In the next article, titled “Enzymatic Assembly of Chitosan-Based Network Polysaccharides and Their Encapsulation and Release of a Fluorescent Dye,” the authors described the preparation of polysaccharide hydrogels by cross-linking chitosan with a cross-linking agent (maltooligosaccharide with a terminal carboxyl group) in a condensation process [Contribution 15]. In the first stage of the study, enzymatic elongation of amylose chains in chitosan network polymers was performed using glucan phosphorylase catalysis. Cross-linking was carried out to obtain modified polysaccharides. Enzymatic polymerization led to the formation of extended gated amylose chains forming double helices. These technologies, using cross-linked chitosan-based polysaccharides, enabled the encapsulation and release of rhodamine B, a fluorescent dye [Contribution 15] [29,30].

## 4. Applications of Polysaccharide (Cellulose, Starch, Glucan and Others)-Based Systems

In addition to chitosan, due to their specific properties, other polysaccharides are often used in many fields, both individually and in mixed systems. This chapter presents the properties and applications of systems based on materials such as cellulose, starch, glucan and others. Liesiene et al. describe studies on the preparation and characterization of cellulose and cellulose–hydroxyapatite scaffolds. These scaffolds are often loaded with anti-inflammatory drugs to reduce the inflammatory response directly at the site of bone/tissue substitute implantation. Additionally, the substances used in these systems can be further selected to improve cell adhesion, proliferation, and differentiation. Based on SEM and microcomputed tomography analyses, the authors demonstrated that the requirements for bone tissue engineering were met. In initial studies, dexamethasone sodium phosphate was used as a filler [Contribution 16] [31,32].

Because the drug was released too quickly, cationic groups were introduced into the cellulose macromolecules to prolong the process. For this purpose, amination was performed using 2-chloro-N,N-diethylethylamine hydrochloride in an alkaline medium. Due to the ionic interactions between the cationic groups in the scaffolds and the anionic groups of the drug molecules, its release was effectively prolonged. Only about 6–7% of the drug was released within one day, and complete release occurred after a week. In addition to prolonging the drug release process, cationic groups in the scaffold structure also facilitated the adsorption of dexamethasone sodium phosphate [Contribution 16].

In the next article, the authors presented the physicochemical characteristics of starch-based systems as a potential carrier for gallic acid. Wettability measurements of various starch types were performed before and after modification using test liquids of different chemical natures via the thin-layer wicking method. High values of the coefficient of determination R2 confirmed the goodness of fit of the linear regression model to describe the relationship between wetting time and the square of the penetration distance for the studied starch systems [Contribution 17] [33,34].

Additionally, the change in free energy (enthalpy) during liquid movement in the tested porous layer was determined. It is well known that the lower the adhesive tension, the easier the wetting process, and, consequently, the adsorption process. Furthermore, the adhesive tension for polar substances refers to the adsorption of hydrophilic substances, while, for nonpolar substances, the adhesive tension refers to the adsorption of hydrophobic substances. The obtained relationships for polar substances are crucial for the adsorption of gallic acid on starch, as it occurs via the formation of donor–acceptor bonds. The authors demonstrated that this type of adsorption is the highest for corn starch compared to other systems studied. This starch therefore has the greatest potential for use as a carrier for gallic acid or, more broadly, for compounds from the polyphenol group [Contribution 17] [33,34].

In a study described in a subsequent article titled “Isolation, Purification, Fractionation, and Hepatoprotective Activity of Polygonatum Polysaccharides”, homogeneous fractions of polysaccharide Polygonatum were obtained [Contribution 18]. During this process, cellulose column, agarose gel and Sephadex chromatography were used. Additionally, the monosaccharide compositions and molecular weights were analyzed. The authors observed that Polygonatum polysaccharides exhibited protective effects against liver damage *in vitro* via both anti-oxidant and anti-inflammatory mechanisms. The results obtained by Wang et al. suggest that Polygonatum polysaccharides may prove to be a promising option for the development of hepatoprotective drugs or functional foods with anti-inflammatory and antioxidant properties [Contribution 18] [35,36].

Kowalczyk et al. described the film-forming properties of α-1,3-glucan systems obtained from mushroom *Laetiporussulphureus* [Contribution 19]. Unlike many natural polymers, glucan is water-insoluble and can be ideal candidate for the production of moisture-resistant films. The study focused on the physico-chemical and biodegradation properties of glucan-made films, which can be used in biodegradable packaging, pharmacy and cosmetics. FTIR and Raman spectroscopy confirmed predominant α-glycosidic linkages in the polysaccharide film structure. Moreover, the films exhibited a semi-crystalline structure and high opacity due to surface roughness resulting from biopolymer coagulation [Contribution 19]. The obtained films showed high moisture content and water solubility, but weak mechanical properties. Water vapor permeability was comparable to other glycerol-plasticized polysaccharide films previously reported. Additionally, films supported the adhesion of soil microorganisms and target bacteria. The authors also proved the high biodegradability of glucan-based films by *Trichoderma harzianum* and endo-/exo-α-1,3-glucanases [Contribution 19] [37,38].

In the article by Jasińska et al. a new approach to the study of antioxidant films was described [Contribution 20]. Two-layer films based on furcellaran and gelatine with added *Phytolacca americana L.* extract (PA) were used as active packaging for African catfish fillets. Films with PA extract were shown to minimize the spoilage of catfish meat and reduce unpleasant odors. However, neither the films nor the PA extract demonstrated antimicrobial activity against the tested microorganism groups, although the tested films exhibited antioxidant activity. Cytotoxicity analysis showed that the PA extract had only a minor effect on prostate epithelial cells, human liver cells, and normal human keratinocytes [Contribution 20] [39,40].

The last two articles in this chapter are typical review articles. The first, by C. Carton and co-workers, describes the physicochemical properties of bioderived oligogalacturonides and their potential applications in crop protection [Contribution 21]. Plants modify their cell walls during pathogen attack. Pectinases, primarily polygalacturonases, play a key role in the controlled hydrolysis of cell wall polysaccharides. This process leads to the formation of oligogalacturonides, which act as key triggers of plant defense mechanisms. Furthermore, it can stimulate innate immunity and enhance resistance to pathogens by modulating the expression of genes involved in the immune response and inducing the production of defense compounds. Pectins can be a natural alternative to conventional phytosanitary products. They can be obtained by chemical, thermal, or enzymatic degradation of pectin-rich plant biomass (from by-products such as citrus peels, apple pomace, and beet pulp). This review: (i) updates knowledge on the methods used in pectin production, (ii) characterizes new sources of pectin production, and (iii) summarizes the potential of pectins as biological control agents [Contribution 21] [41,42].

The second review article, by S. Akhmetova et al., describes various types of organic–inorganic composites applicable for catalytic applications [Contribution 22]. Polysaccharides offer several advantages over synthetic polymers and have recently been used to design catalysts in organic synthesis. This review discusses the current applications of typical polysaccharides (chitosan, starch, pectin, cellulose, and hydroxyethylcellulose) and their composites in catalysis. The article is divided into a few main sections characterizing different groups of nanocomposites: (i) chitosan-based, (ii) pectin-based (iii) cellulose-based or (iv) starch-based nanocomposites. The authors demonstrated that by modifying polysaccharides, polymers with specific properties can be obtained, thus expanding the scope of biocomposites for catalytic applications [Contribution 22] [43,44].

## 5. Theoretical Investigation of Polysaccharide-Based Systems

Due to their structural complexity and wide spectrum of applications, research on polysaccharides requires thorough analysis, both theoretical and practical. Therefore, this chapter describes theoretical studies of the properties of polysaccharide systems, primarily chitosan-based ones. One such property is the degree of deacetylation, *DDA*, which corresponds to the percentage of D-glucosamine monomers in the chitosan structure. The *DDA* value determines the possibility and number of different chemical modifications leading to the creation of new materials. Potentiometric titration, one of the simplest, most readily available, and cheapest methods, is often used to determine *DDA*. However, this method is characterized by low precision—for example, in the case of modified chitosan resins. This is largely because the equation used to calculate *DDA* does not take into account the molecular weight of the monomer units. In this study, the authors introduced a new equation, specifically for modified chitosan containing three different types of monomers. This equation, used for naphthalene–chitosan resins, is closer to the actual structure of the polymer chains and yields higher *DDA* values than the classical equation [Contribution 23] [45,46].

As noted above, chitosan-based materials have versatile applications, from biotechnology to the pharmaceutical industry. The degree and mode of acetylation along the chitosan chain modulate its biological and physicochemical properties. However, the molecular mechanism remains poorly understood. In the article by A. Romany and co-workers, the authors presented *de novo* molecular dynamics (MD) simulations to investigate the self-assembly process of chitosan with varying degrees and patterns of acetylation. They demonstrated that 10-mer chitosan chains with 50% acetylation assemble to form ordered nanofibrils composed primarily of antiparallel chains. It is worth emphasizing that the simulations were consistent with diffraction data for deacetylated chitosan [Contribution 24] [47,48].

It was found that, regardless of the acetylation pattern, intermolecular hydrogen bonds mediate sheet formation, while interactions of water molecules stabilize sheet stacking. Furthermore, the acetylated species involved in the formation of strong intermolecular hydrogen bonds may support the notion that increased acetylation reduces chitosan solubility. In summary, the authors’ theoretical considerations and improved molecular mechanics parameters bring us closer to understanding the role of acetylation at the atomic level in modulating chitosan physicochemical properties. This, in turn, is very helpful in the design of chitosan-based materials using different degrees and patterns of acetylation [Contribution 24] [47,48].

Finally, we would like to thank all the authors who submitted manuscripts to our special issue “Chitosan, Chitosan Derivatives, Polysaccharides, and Their Applications—Issue 2” for their high-quality papers. We also extend our sincere thanks to current and future readers. We hope that this issue will become a platform for dialogue among scientists working on polysaccharide systems, particularly the use of chitosan/chitosan derivatives, as well as new perspectives on their diverse applications.

## References

[1] Gheorghita R., Anchidin-Norocel L., Filip R., Dimian M., Covasa M. (2021). Applications of Biopolymers for Drugs and Probiotics Delivery. Polymers.

[2] Yao Y., Xu B. (2022). Skin Health Promoting Effects of Natural Polysaccharides and Their Potential Application in the Cosmetic Industry. Polysaccharides.

[3] Choukaife H., Seyam S., Alallam B., Doolaanea A.A., Alfatama M. (2022). Current Advances in Chitosan Nanoparticles Based Oral Drug Delivery for Colorectal Cancer Treatment. Int. J. Nanomed..

[4] Bekmukhametova A., Antony A., Halliday C., Chen S., Ho C.-H., Uddin M.M.N., Longo L., Pedrinazzi C., George L., Wuhrer R. (2024). Rose Bengal–Encapsulated Chitosan Nanoparticles for the Photodynamic Treatment of *Trichophyton* Species. Photochem. Photobiol..

[5] Wang M., Wu Y., Li G., Lin Q., Zhang W., Liu H., Su J. (2024). Articular Cartilage Repair Biomaterials: Strategies and Applications. Mater. Today Bio..

[6] Ghobadi F., Mohammadi M., Kalantarzadeh R., Lotfi E., Borhan S., Chauhan N.P.S., Salehi G., Simorgh S. (2025). Advanced 3D-Printed Multiphasic Scaffold with Optimal PRP Dosage for Chondrogenesis of BM-MSCs in Osteochondral Tissue Engineering. arXiv.

[7] Klimek K., Ginalska G. (2020). Proteins and Peptides as Important Modifiers of the Polymer Scaffolds for Tissue Engineering Applications—A Review. Polymers.

[8] Norahan M.H., Pedroza-González S.C., Sánchez-Salazar M.G., Alvarez M.M., de Santiago G.T. (2023). Structural and Biological Engineering of 3D Hydrogels for Wound Healing. Bioact. Mater..

[9] Das A., Ghosh S., Pramanik N. (2024). Chitosan Biopolymer and its Composites: Processing, Properties and Applications—A Comprehensive Review. Hybrid Adv..

[10] Kanwal S., Bibi S., Haleem R., Waqar K., Mir S., Maalik A., Sabahat S., Hassan S., Awwad N.S., Ibrahium H.A. (2024). Functional Potential of Chitosan-Metal Nanostructures: Recent Developments and Applications. Int. J. Biol. Macromol..

[11] Zhou P., Zhang Z., Mo F., Wang Y. (2024). A Review of Functional Hydrogels for Flexible Chemical Sensors. Adv. Sens. Res..

[12] Sarac B., Yücer S., Sahin H., Unal M., Ciftci F. (2024). Wearable and Implantable Bioelectronic: Biosensing Contact Lens and Applications. Chem. Eng. J..

[13] Tesco G., Lomoio S. (2022). Pathophysiology of Neurodegenerative Diseases: An Interplay among Axonal Transport Failure, Oxidative Stress, and Inflammation?. Semin. Immunol..

[14] Xu S., Wen S., Yang Y., He J., Yang H., Qu Y., Zeng Y., Zhu J., Fang F., Song H. (2024). Association Between Body Composition Patterns, Cardiovascular Disease, and Risk of Neurodegenerative Disease in the UK Biobank. Neurology.

[15] Saldaña-Mendoza S.A., Pacios-Michelena S., Palacios-Ponce A.S., Chávez-González M.L., Aguilar C.N. (2023). Trichoderma as a Biological Control Agent: Mechanisms of Action, Benefits for Crops and Development of Formulations. World J. Microbiol. Biotechnol..

[16] Negri A., Pezzali G., Pitton S., Piazzoni M., Gabrieli P., Lazzaro F., Mastrantonio V., Porretta D., Lenardi C., Caccia S. (2023). MosChito Rafts as a Promising Biocontrol Tool Against Larvae of the Common House Mosquito, Culex pipiens. PLoS ONE.

[17] Du B., Haensch R., Alfarraj S., Rennenberg H. (2024). Strategies of Plants to Overcome Abiotic and Biotic Stresses. Biol. Rev..

[18] Oyebamiji Y.O., Adigun B.A., Shamsudin N.A.A., Ikmal A.M., Salisu M.A., Malike F.A., Lateef A.A. (2024). Recent Advancements in Mitigating Abiotic Stresses in Crops. Horticulturae.

[19] Dukarski W., Rykowska I., Krzyżanowski P., Gonsior M. (2024). Flame Retardant Additives Used for Polyurea-Based Elastomers—A Review. Fire.

[20] Shen J., Liang J., Lin X., Lin H., Yu J., Wang S. (2022). The Flame-Retardant Mechanisms and Preparation of Polymer Composites and Their Potential Application in Construction Engineering. Polymers.

[21] Wang X., Wei W., Guo Z., Liu X., Liu J., Bing T., Yu Y., Yang X., Cai Q. (2024). Organic–Inorganic Composite Hydrogels: Compositions, Properties, and Applications in Regenerative Medicine. Biomater. Sci..

[22] Oliveira L.R., Nassar E.J., da Silva Barud H., Silva J.M., Rocha L.A. (2022). Polymer and Biopolymer Organic-Inorganic Composites Containing Mixed Oxides for Application in Energy up- and Down-conversion. Opt. Mater..

[23] Berdimurodov E., Eliboyev I., Berdimuradov K., Kholikov A., Akbarov K., Dagdag O., Rbaa M., El Ibrahimi B., Verma D.K., Haldhar R. (2022). Green β-Cyclodextrin-Based Corrosion Inhibitors: Recent Developments, Innovations and Future Opportunities. Carbohydr. Polym..

[24] Nawaz M., Ahmad S., Taryba M.G., Montemor M.F., Kahraman R., Shakoor R.A. (2024). Improvement in Inhibition Performance of Anti-Corrosion Coatings Using Polyolefin Matrix Embedded with Modified TiO_2_ Nanoparticles. Prog. Org. Coat..

[25] Przykaza K., Jurak M., Kalisz G., Mroczka R., Wiącek A.E. (2023). Characteristics of Hybrid Bioglass-Chitosan Coatings on the Plasma Activated PEEK Polymer. Molecules.

[26] Szafran K., Jurak M., Mroczka R., Wiącek A.E. (2023). Preparation and Surface Characterization of Chitosan-Based Coatings for PET Materials. Molecules.

[27] Jankowska K., Su Z., Zdarta J., Jesionowski T., Pinelo M. (2022). Synergistic Action of Laccase Treatment and Membrane Filtration during Removal of Azo Dyes in an Enzymatic Membrane Reactor Upgraded with Electrospun Fibers. J. Hazard. Mater..

[28] Strebel A., Behringer M., Hilbig H., Machner A., Helmreich B. (2024). Anionic Azo Dyes and Their Removal from Textile Wastewater through Adsorption by Various Adsorbents: A Critical Review. Front. Environ. Eng..

[29] Papagiannopoulos A., Sotiropoulos K. (2022). Current Advances of Polysaccharide-based Nanogels and Microgels in Food and Biomedical Sciences. Polymers.

[30] Nakamichi A., Kadokawa J. (2022). Fabrication of Chitosan-Based Network Polysaccharide Nanogels. Molecules.

[31] Iglesias-Mejuto A., Magariños B., Ferreira-Gonçalves T., Starbird-Pérez R., Álvarez-Lorenzo C., Reis C.P., Ardao I., García-González C.A. (2024). Vancomycin-Loaded Methylcellulose Aerogel Scaffolds for Advanced Bone Tissue Engineering. Carbohydr. Polym..

[32] Majrashi M., Kotowska A., Scurr D., Hicks J.M., Ghaemmaghami A., Yang J. (2023). Sustained Release of Dexamethasone from 3D-Printed Scaffolds Modulates Macrophage Activation and Enhances Osteogenic Differentiation. ASC Appl. Mater. Interfaces.

[33] Nájera-Martínez E.F., Flores-Contreras E.A., Araújo R.G., Iñiguez-Moreno M., Sosa-Hernández J.E., Iqbal H.M.N., Pastrana L.M., Melchor-Martínez E.M., Parra-Saldívar R. (2023). Microencapsulation of Gallic Acid Based on a Polymeric and PH-Sensitive Matrix of Pectin/Alginate. Polymers.

[34] Lei W., Liang J., Tan P., Yang S., Fan L., Han M., Li H., Gao Z. (2022). Preparation of Edible Starch Nanomaterials for the Separation of Polyphenols from Fruit Pomace Extract and Determination of Their Adsorption Properties. Int. J. Biol. Macromol..

[35] Li H., Xu G., Yuan G. (2022). *Armillaria mellea* Effects of an Polysaccharide on Learning and Memory of D-Galactose-Induced Aging Mice. Front. Pharmacol..

[36] Gao L., Du B., Ma Q., Ma Y., Yu W., Li T., Liu Y., Yuan G. (2024). Multiplex-PCR Method Application to Identify Duck Blood and its Adulterated Varieties. Food Chem..

[37] Łupina K., Kowalczyk D., Zięba E., Kazimierczak W., Mężyńska M., Basiura-Cembala M., Wiącek A.E. (2019). Edible Films Made from Blends of Gelatin and Polysaccharide-Based Emulsifiers—A Comparative Study. Food Hydrocoll..

[38] Rusetskaya V., Różalska S., Słaba M., Bernat P. (2024). Degradation Ability of *Trichoderma spp.* in the Presence of Poly(butylene adipate-co-terephthalate) microparticles. Int. Biodeterior. Biodegrad..

[39] Abelti A.L., Teka T.A. (2022). Development and Characterization of Biodegradable Polymers for Fish Packaging Applications. J. Packag. Technol. Res..

[40] Birania S., Kumar S., Kumar N., Attkan A.K., Panghal A., Rohilla P., Kumar R. (2022). Advances in Development of Biodegradable Food Packaging Material from Agricultural and Agro-industry Waste. J. Food Process Eng..

[41] Silva-Sanzana C., Zavala D., Moraga F., Herrera-Vásquez A., Blanco-Herrera F. (2022). Oligogalacturonides Enhance Resistance against Aphids through Pattern-Triggered Immunity and Activation of Salicylic Acid Signaling. Int. J. Mol. Sci..

[42] Martínez-Gómez S., Fernández-Bautista M., Rivas S., Yáñez R., Alonso J.L. (2023). Recent Advances in the Production of Oligogalacturonides and Their Biological Properties. Food Funct..

[43] Díaz-Montes E. (2022). Polysaccharides: Sources, Characteristics, Properties, and Their Application in Biodegradable Films. Polysaccharides.

[44] Wu Q., Cheng N., Fang D., Wang H., Rahman F., Hao H., Zhang Y. (2024). Recent Advances on Application of Polysaccharides in Cosmetics. J. Dermatol. Sci. Cosmet. Technol..

[45] Chen Q., Qi Y., Jiang Y., Quan W., Luo H., Wu K., Li S., Ouyang Q. (2022). Progress in Research of Chitosan Chemical Modification Technologies and Their Applications. Mar. Drugs.

[46] Omer A.M., Eweida B.Y., Tamer T.M., Soliman H.M.A., Mohamed A.S., Zaatot A.A., Mohy-Eldin M.S. (2021). Removal of Oil Spills by Novel Developed Amphiphilic Chitosan-g-citronellal Schiff base Polymer. Sci. Rep..

[47] Romany A., Payne G.F., Shen J. (2023). Mechanism of the Temperature-Dependent Self-assembly and Polymorphism of Chitin. Chem. Mater..

[48] Sreekumar S., Wattjes J., Niehues A., Mengoni T., Mendes A.C., Morris E.R., Goycoolea F.M., Moerschbacher B.M. (2022). Biotechnologically Produced Chitosans with Nonrandom Acetylation Patterns Differ From Conventional Chitosans in Properties and Activities. Nat. Commun..

